# Intravoxel incoherent motion DWI of the pancreatic adenocarcinomas: monoexponential and biexponential apparent diffusion parameters and histopathological correlations

**DOI:** 10.1186/s40644-017-0114-8

**Published:** 2017-04-28

**Authors:** Chao Ma, Yanjun Li, Li Wang, Yang Wang, Yong Zhang, He Wang, Shiyue Chen, Jianping Lu

**Affiliations:** 1 Department of Radiology, Changhai Hospital of Shanghai, The Second Military Medical University, No.168 Changhai Road, Shanghai, 200433 China; 2 Department of Pathology, Changhai Hospital of Shanghai, The Second Military Medical University, No.168 Changhai Road, Shanghai, China; 3 MR Group, GE Healthcare, No. 1 Huatuo Road, Shanghai, China

**Keywords:** IVIM, Apparent diffusion coefficient, Pancreatic cancer, DWI, Biexponential apparent diffusion

## Abstract

**Background:**

To investigate the associations between the diffusion parameters obtained from multiple-b-values diffusion weighted imaging (DWI) of pancreatic ductal adenocarcinoma (PDAC) and the aggressiveness and local stage prediction, and assess the values of the quantitative parameters for the discrimination of tumors from healthy pancreas.

**Methods:**

Fifty-one patients with surgical pathology-proven PDAC (size, 35 ± 12 mm) and fifty-seven healthy volunteers were enrolled. Diffusion parameters including monoexponential apparent diffusion coefficient (ADC_b_ and ADC_total_) and biexponential intravoxel incoherent motion (IVIM) parameters (ADC_slow_, ADC_fast_ and f) based on 9 b-values (0 to 1000s/mm^2^) DWI were calculated for the lesions and the healthy pancreas. These parameters were compared by grades of differentiation, lymph node status, tumor stage and location. The diagnostic performances were calculated and compared by using the receiver operating characteristic curves (ROC) analyses.

**Results:**

There was no statistically significant difference in ADC_b_, ADC_total_, ADC_slow_, ADC_fast_ or f between PDAC stage T1/T2 and stage T3/T4 or moderately differentiated versus poorly differentiated PDAC (*p* = 0.060-0.941). In addition, no significant differences were observed for the quantitative parameters between tumors located in the pancreatic head versus other pancreatic regions (*p =* 0.203-0.954) or between tumors with and without metastatic peri-pancreatic lymph nodes (*p =* 0.313-0.917). ADC_25-600_, ADC_1000_, ADC_total_ and ADC_fast_ were significantly lower for PDAC compared the healthy pancreas (all p < 0.05). ROC analyses showed the area under curve for ADC_20_ was the largest (0.911) to distinguish PDAC from normal pancreas (cut-off value, 5.58 × 10^−3^mm^2^/s) and had the highest combined sensitivity (89.5%) and specificity (82.4%).

**Conclusions:**

Multiple-b-values DWI derived monoexponential and biexponential parameters of PDAC do not exhibit significance dependence on tumor grade or tumor characteristics. ADC_20_ provided the best accuracy for differentiating PDAC from healthy pancreas in the study.

## Background

Pancreatic cancer accounts for about 3% of all cancer cases [1]. It is one of the few cancers which have shown little improvement in survival rate over the past 40 years [2]. Diagnosis of the early stages of pancreatic cancer is difficult even with powerful imaging techniques such as computed tomography (CT), magnetic resonance imaging (MRI), transabdominal and endoscopic ultrasonography (EUS) and endoscopic retrograde cholangiopancreatography (ERCP) [3, 4]. About 74% patients with pancreatic cancer die within the first year of diagnosis [1].

Diffusion-weighted magnetic resonance imaging (DWI) is the only noninvasive technique exploring the microscopic mobility of water molecules in the tissues without contrast administration. The diffusion of water molecules in the human body can be quantified by apparent diffusion coefficient (ADC) based on DWI [5]. Recent technique advancements allow DWI and ADC measurements to be increasingly used in the diagnosis of abdominal diseases [6–8]. Several studies have demonstrated significantly lower ADC in pancreatic cancer than in benign pancreas tissue [9–23]. There is still diagnostic challenge as described by Fukukura et al [13], also the published range of ADC values for both normal and neoplastic tissues varied dramatically as reported in different studies [9–23]. Recently, the role of ADC values in predicting adverse pathological features of pancreatic cancer were reported [12, 24, 25]. However, conflicting results have been described: significant association [24] and lack of association [12, 25] between the ADC and pathological grade of pancreatic cancer were reported. These reports, however, used only two b values (0, 500 or 800 s/mm^2^) to measure ADC, which is influenced not only by the structures of the tissue, but also by the microcirculation of blood in the capillary network. Ideally, multiple-b-values DWI with intravoxel incoherent motion (IVIM) model should be set up for the separate estimation of tissue perfusion and diffusivity [26].

The objective of this study was to investigate potential associations between the DWI-derived IVIM parameters such as ADC_fast_ (pseudo-diffusion coefficient), ADC_slow_ (the tissue coefficient), f (perfusion fraction) and the commonly used DWI-derived monoexponential ADC in pancreatic cancer and the tumor grade as well as tumor characteristics including lymph node status, tumor stage and location [12]. In addition, we also investigated the values of multiple-b-values DWI derived parameters for the discrimination of tumors from healthy pancreas.

## Methods

### Subjects

**Fig. 1:**
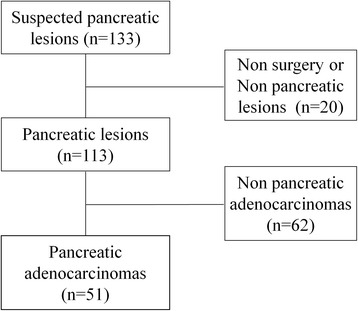
Flowchart of study patients’ inclusion process

This prospective study was approved by our Institutional Review Board. Signed written informed consent was obtained from all participants before MRI examinations. We enrolled 133 patients with a suspect pancreatic mass seen in a CT or US between May 2011 and June 2013 from the inpatients (Fig. 1). Among them, 113 patients received surgery within 7 days after the time of inclusion in our study. 51 patients were pancreatic ductal adenocarcinomas (PDAC) (27 men, 24 women; mean age 59.6 years; range 36-76 years) with histopathologic diagnoses. We also enrolled 57 healthy volunteers (36 men and 21 women; mean age 45.0 years; range 21-68 years) as the control group. Exclusion criteria for the healthy volunteers included diseases which might affect normal pancreatic function, such as pancreatic disease, severe fatty liver and hepatic cirrhosis history.

### Image Acquisition

All examinations were performed on a 3.0-Tesla MR (Signa HDxt V16.0, GE Healthcare, Milwaukee, USA) with an eight-element phased array coil. All the participants underwent MRI protocols including transverse respiratory triggered single-shot echo-planar DWI (weighted along three orthogonal gradient directions) with b values of 0, 20, 50, 100, 200, 400, 600, 800 and 1000 s/mm^2^. Selective presaturation with inversion recovery (SPIR) was used for fat saturation; two saturation slabs were fixed on the A/P direction to reduce potential motion artifacts. The main scan parameters of MRI sequences were listed in the Table 1. Only the 51 patients underwent contrast-enhanced liver acceleration volume acquisition (LAVA) which was performed with Gadopentetate Dimeglumine injection (physiological saline, 10-15ml; media, 0.1-0.15 mmol/kg; injection rate, 2-3 ml/s) at the end of the study.

**Table 1:** The main parameters of MR sequences

Sequences	TR/TE (ms)	FOV (mm)	Matrix	Thickness/gap (mm)	Flip angle (^0^)	slices	NEX	Band width (KHz)	Acceleration factor
2D Single-Shot Fast Spin Echo, SSFSE (MRCP)	7000/1221	300 × 300	288 × 288	64/0	-	6	0.92	31.3	-
Axial Fast Spin Echo, FSE (T2WI)	6316/72	360 ~ 400	320 × 192	5/1	90	20	2	83.3	2
Axial Single-Shot Echo Planar Imaging, ss DWEPI (DWI)	3333/66.8	360 ~ 400	192 × 160	5/1	90	20	4	250	2
3D fat-suppressed Gradient Echo, 3D GRE (LAVA)	2.5/1.1	440 × 418	256 × 180	5/0	11	76	0.71	125	2

### Data analysis

DWI-data were processed using a standard software package (Function 6.3.1e, GE AW VolumeShare 2, GE Healthcare, Milwaukee, USA). The multiple-b-values DWI derived parameters were calculated for all slices voxel-by-voxel with the following three approaches, which have been presented in our previously study in details [27]:

1. Direct calculation of the ADC_b_ using only two b-values (zero and non-zero):
  $$ {\mathrm{ADC}}_{\mathrm{b}}=\frac{1}{\mathrm{b}} \ln \left(\frac{{\mathrm{S}}_0}{\mathrm{S}}\right) $$
2. The ADC_total_ calculation by monoexponential fitting to the equation using all b-values:
  $$ \frac{\mathrm{S}}{{\mathrm{S}}_0}= \exp \left(-\mathrm{b}\times {\mathrm{ADC}}_{\mathrm{total}}\right) $$
3. Biexponential fitting on IVIM model gave ADC_fast_, ADC_slow_, f according to the following equation:
  $$ \frac{\mathrm{S}}{{\mathrm{S}}_0}=\mathrm{f} \exp \left(-\mathrm{b}\times {\mathrm{ADC}}_{\mathrm{fast}}\right)+\left(1-\mathrm{f}\right) \exp \left(-\mathrm{b}\times {\mathrm{ADC}}_{\mathrm{slow}}\right) $$

### Image Analysis

**Fig. 2:**
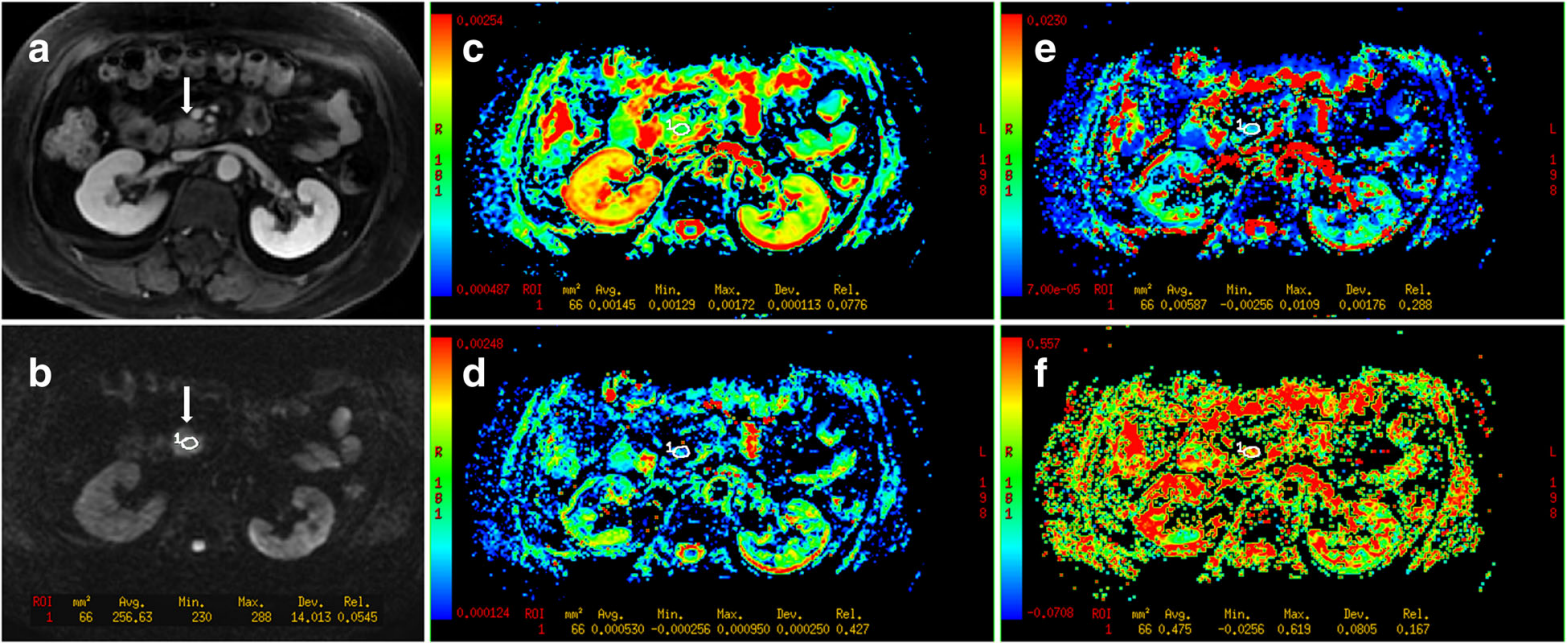
Images from a 67-year-old woman with a moderate differentiated pancreatic ductal adenocarcinoma on the head of the pancreas. (A) Axial contrast-enhanced T1-weighted image shows hypovascularity of the mass (arrow). (B) The nodule is detected on the DW image (b = 1000 s/mm^2^) with clear hyperintensity relative to the remainder of the pancreas (arrow). (C-F) The calculated ADC_total_, ADC_slow_, ADC_fast_ and f -maps, respectively (measurements of the values of ROI were showed in the images)

Quantitative analysis of DWI was performed by two readers (6-year and 4-year experience) in consensus. All available data, including the ADC_b_-, ADC_total_-, ADC_slow_-, ADC_fast_-, f maps and DWI images, were loaded in the software in conjunction. Region of interests (ROIs) were drawn on multiple slices of the images of b_1000_ and were directly co-localized on the diffusion parameters maps, respectively. For the tumor diffusion parameters measurements, mean values of ADC_20-1000_, ADC_total_, ADC_fast_, ADC_slow_ and f were calculated from an oval ROI (mean 118 mm^2^; range 55 - 308 mm^2^), which was placed on the solid portion of the tumor (Fig. 2), avoiding pancreatic ducts and cystic lesions by referring to other MRI images such as T2WI or LAVA. In the healthy cases, conventional MR sequences including T2WI, LAVA images did not show any diffuse parenchymal abnormalities. No substantial distortion artifacts were visible in the pancreas. The mean values of ADC_20-1000_, ADC_total_, ADC_fast_, ADC_slow_ and f for normal pancreatic tissue were derived from an oval ROI (mean 64.5 mm^2^; range 35-108 mm^2^), which was drawn in the head of the pancreas and kept away from the border of the pancreas to prevent partial volume effect. An effort was made to avoid pancreatic duct, vessels, and the common bile duct.

### Histological Analysis

Histopathological analyses were performed by a pathologist with 12 years of experience specifically for pancreatic diseases. Surgically resected specimens were used for the pathological evaluation of all tumors, which were subcategorized as well, moderately, and poorly differentiated adenocarcinomas according to the classification system of the World Health Organization (WHO) [28] and practical grading scheme for pathology [29] in the current study. Meantime, pathologically determined tumor size, T stage, and nodal status (whether metastatic peri-pancreatic lymph nodes were identified) were recorded for each case.

### Statistical analysis

Statistical analyses were performed using the SPSS software for windows (Version 16.0, SPSS Inc., Chicago, IL, USA). The extracted parameters values were tested for significant differences between patients with PDAC of poorly and moderately differentiated; stage T3/T4 and stage T1/T2 tumors (given the infrequency of stage T1 and T4 tumors); tumors with and without metastatic peri-pancreatic lymph nodes; and tumors located in the pancreatic head versus body or tail using a Mann-Whitney U test, which also was used to compare the multi-b-values DWI derived parameters between pancreatic tumors and healthy pancreas. Spearman-rank correlations were used to assess the relationship between these quantitative parameters and tumor size. The comparison of mean ADC_b_ values of the PDAC or the healthy pancreas among different b values was analyzed using Friedman tests. For the multiple comparisons of ADC values, post-hoc analyses were performed with Wilcoxon signed-rank tests and a Bonferroni correction applied. The statistical significance threshold of the Friedman test was set at a p-value below 0.05, while at a p-value below 0.0018 (0.05/28) for post hoc tests. In addition, receiver operating characteristics (ROC) analyses were used to identify the diagnostic performances of the multiple-b-values DWI derived parameters to distinguish pancreatic cancer from healthy pancreas tissue. A *P*-value of less than 0.05 was considered to indicate a statistically significant difference.

## Results

### Tumors

Based on the WHO classification criteria, 14 patients with poorly differentiated adenocarcinoma and 37 patients with moderately differentiated tumors were identified. The 51 tumors had a mean maximum lesion diameter at histopathological analyses of 35 ± 12 mm (range 15-90 mm). Among the 51 tumors, 30 (58.8%) were located in the pancreatic head; 38 (74.5%) were stage T3/T4 and 29 (56.9%) had metastatic peri-pancreatic lymph nodes.

### Comparisons of IVIM DWI parameters between PDAC and healthy pancreas

**Fig. 3:**
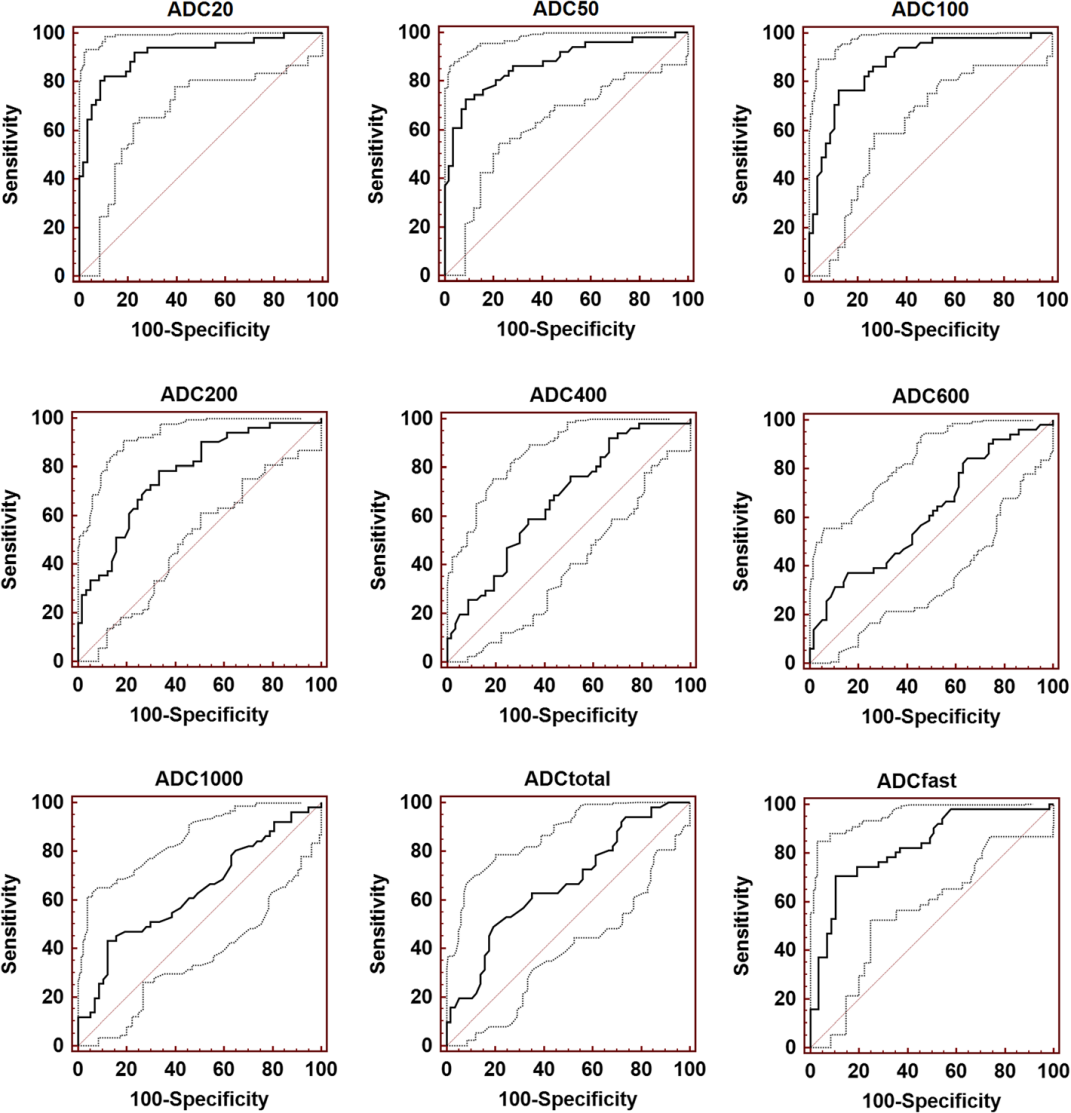
ROC-curves for differentiating pancreatic cancer from healthy pancreas of ADC_20-600_, ADC_1000_, ADC_total_, and ADC_fast_. ADC_20_ revealed significantly higher AUC than other multiple-b-values DWI derived parameters

The Friedman tests results demonstrated significant declines of the mean ADCs of the monoexponential DWI from b_20_ to b_1000_ for the PDAC or the healthy pancreatic tissue (both P < 0.001, Table 2). The mean ADC_20-600_, ADC_1000_, ADCt_otal_, ADC_fast_ values were significantly lowers for PDAC than for healthy pancreas. The diagnostic performances of ADC_20-600_, ADC_1000_, ADCt_otal_, ADC_fast_ for differentiating PDAC form healthy pancreas was shown in Fig. 3 and the ROC analyses results were summarized in Table 3. The largest area under curve (AUC) was 0.911 for ADC_20_ with a cut-off value of 5.58 × 10^−3^ mm^2^/s, and ADC_20_ also had the highest combined sensitivity (89.5%) and specificity (82.4%).

**Table 2:** Comparisons of multi-b-value DWI derived parameters (mean ± standard deviation) of healthy pancreas and pancreatic adenocarcinoma

Parameters	Pancreatic cancer	Healthy pancreas	*P*
ADC_20_ (×10^−3^mm^2^/s)	4.08 ± 2.19	9.01 ± 3.76	*0.000*
ADC_50_ (×10^−3^mm^2^/s)	2.62 ± 1.57	5.19 ± 2.07	*0.000*
ADC_100_ (×10^−3^mm^2^/s)	1.96 ± 0.92	3.72 ± 1.60	*0.000*
ADC_200_ (×10^−3^mm^2^/s)	1.86 ± 0.65	2.61 ± 0.82	*0.000*
ADC_400_ (×10^−3^mm^2^/s)	1.72 ± 0.43	2.03 ± 0.52	*0.003*
ADC_600_ (×10^−3^mm^2^/s)	1.61 ± 0.43	1.76 ± 0.38	*0.037*
ADC_800_ (×10^−3^mm^2^/s)	1.49 ± 0.33	1.61 ± 0.34	0.177
ADC_1000_ (×10^−3^mm^2^/s)	1.20 ± 0.28	1.31 ± 0.25	*0.016*
ADC_total_ (×10^−3^mm^2^/s)	1.38 ± 0.26	1.56 ± 0.30	*0.003*
ADC_fast_ (×10^−3^mm^2^/s)	6.39 ± 5.55	14.05 ± 8.31	*0.000*
ADC_slow_ (×10^−3^mm^2^/s)	0.84 ± 0.32	0.92 ± 0.26	0.235
f (%)	0.42 ± 0.15	0.39 ± 0.12	0.212

ADC indicates apparent diffusion coefficient; IVIM, intravoxel incoherent motion; SD, standard deviation; *P* < 0.05 was considered to indicate a statistically significant difference

**Table 3:** Results from the ROC analyses for the 9 parameters to distinguish between pancreatic adenocarcinoma and healthy pancreas

Parameters	Optimal cutoff values (×10^−3^mm^2^/s)	AUC	Sensitivities (%)	Spectificities (%)	PPV (%)	NPV (%)	ACC (%)
ADC_20_	5.58	0.911	0.895	0.824	0.820	0.898	0.858
ADC_50_	3.06	0.874	0.912	0.725	0.748	0.902	0.813
ADC_100_	2.37	0.876	0.877	0.765	0.770	0.874	0.818
ADC_200_	2.19	0.771	0.667	0.784	0.734	0.725	0.729
ADC_400_	1.91	0.667	0.491	0.765	0.651	0.627	0.636
ADC_600_	1.41	0.616	0.842	0.373	0.546	0.725	0.594
ADC_1000_	1.09	0.634	0.877	0.431	0.580	0.797	0.642
ADC_total_	1.36	0.664	0.807	0.490	0.586	0.739	0.640
ADC_fast_	5.86	0.828	0.895	0.706	0.731	0.883	0.795

ROC, operating characteristic curve; AUC, area under curve; ADC, apparent diffusion coefficient; PPV, positive predictive value; NPV, negative predictive value; ACC, accuracy

### Association between IVIM DWI parameters and tumor grade

The mean ADC values in PDAC with moderate differentiation were similar to those with poor differentiation at b_20-1000_ (*p =* 0.460-0.941). In addition, no significant differences were observed between the two groups for ADC_total_ (*P* = 0.720), ADC_slow_ (*P* = 0.658), ADC_fast_ (*P* = 0.326) and f (*P* = 0.941). The results were summarized in Table 4.

**Table 4:** Comparisons of multi-b-value DWI derived parameters (mean ± standard deviation) of pancreatic adenocarcinoma with tumor characteristics

Parameters	Grades of differentiation			Tumor locations			Tumor grades			lymph node status		
	well/moderately differentiated	poorly differentiated	*P*	Head	Elsewhere in pancreas	*P*	T1/T2	T3/T4	*P*	Present	Absent	*P*
ADC_20_ (×10^−3^mm^2^/s)	4.15 ± 2.24	3.92 ± 2.13	0.849	4.05 ± 2.12	4.13 ± 2.35	0.901	4.24 ± 2.78	4.06 ± 2.04	0.846	3.97 ± 2.37	4.17 ± 2.09	0.419
ADC_50_ (×10^−3^mm^2^/s)	2.62 ± 1.69	2.62 ± 1.23	0.665	2.44 ± 1.36	2.87 ± 1.83	0.358	3.04 ± 1.33	2.50 ± 1.65	0.098	2.64 ± 1.79	2.60 ± 1.40	0.897
ADC_100_ (×10^−3^mm^2^/s)	2.03 ± 1.01	1.77 ± 0.65	0.499	1.89 ± 0.71	2.06 ± 1.18	0.954	2.19 ± 0.81	1.88 ± 0.96	0.107	1.95 ± 0.96	1.97 ± 0.92	0.775
ADC_200_ (×10^−3^mm^2^/s)	1.89 ± 0.74	1.77 ± 0.29	0.460	1.86 ± 0.51	1.85 ± 0.82	0.599	2.10 ± 0.44	1.79 ± 0.70	0.060	1.93 ± 0.79	1.81 ± 0.53	0.827
ADC_400_ (×10^−3^mm^2^/s)	1.73 ± 0.49	1.68 ± 0.19	0.768	1.69 ± 0.35	1.76 ± 0.52	0.811	1.80 ± 0.27	1.70 ± 0.46	0.496	1.74 ± 0.43	1.70 ± 0.43	0.732
ADC_600_ (×10^−3^mm^2^/s)	1.63 ± 0.49	1.57 ± 0.23	0.941	1.53 ± 0.28	1.73 ± 0.57	0.320	1.73 ± 0.29	1.59 ± 0.46	0.226	1.65 ± 0.51	1.58 ± 0.36	0.662
ADC_800_ (×10^−3^mm^2^/s)	1.51 ± 0.36	1.44 ± 0.19	0.486	1.43 ± 0.26	1.57 ± 0.40	0.235	1.60 ± 0.27	1.46 ± 0.34	0.256	1.51 ± 0.29	1.47 ± 0.35	0.917
ADC_1000_ (×10^−3^mm^2^/s)	1.22 ± 0.31	1.14 ± 0.17	0.506	1.16 ± 0.23	1.25 ± 0.32	0.203	1.21 ± 0.17	1.20 ± 0.30	0.880	1.18 ± 0.29	1.21 ± 0.27	0.337
ADC_total_ (×10^−3^mm^2^/s)	1.38 ± 0.29	1.35 ± 0.16	0.720	1.34 ± 0.22	1.43 ± 0.31	0.450	1.47 ± 0.20	1.36 ± 0.27	0.399	1.37 ± 0.19	1.38 ± 0.30	0.387
ADC_fast_ (×10^−3^mm^2^/s)	6.98 ± 6.28	4.81 ± 2.38	0.326	6.87 ± 6.49	5.70 ± 3.88	0.612	8.89 ± 9.15	5.58 ± 3.74	0.154	5.63 ± 3.6	6.96 ± 6.67	0.634
ADC_slow_ (×10^−3^mm^2^/s)	0.86 ± 0.32	0.81 ± 0.33	0.658	0.80 ± 0.31	0.91 ± 0.33	0.343	0.86 ± 0.37	0.84 ± 0.31	0.689	0.80 ± 0.36	0.88 ± 0.29	0.313
f (%)	0.42 ± 0.15	0.42 ± 0.14	0.941	0.41 ± 0.16	0.43 ± 0.13	0.653	0.43 ± 0.17	0.42 ± 0.14	0.854	0.43 ± 0.16	0.41 ± 0.14	0.562

ADC indicates apparent diffusion coefficient; IVIM, intravoxel incoherent motion; SD, standard deviation

### Association between IVIM DWI parameters and tumor characteristics

There were no significant correlations between multiple-b-values DWI derived parameters values and tumor size (*P* = 0.195-0.986). There was no statistically significant difference in all of the multi-b-values DWI derived parameters between PDAC stage T1/T2 and stage T3/T4. (*p* = 0.060-0.880). In addition, all of the quantitative parameters were not significantly different between tumors located in the pancreatic head versus other pancreatic regions (*p* = 0.203-0.954) or between tumors with and without metastatic peri-pancreatic lymph nodes (*p* = 0.313-0.917).

## Discussion

Our study showed that multiple-b-values DWI derived parameters including ADC_20-600_, ADC_1000_, ADCt_otal_, ADC_fast_ might be useful markers to distinguish PDAC from healthy pancreas, and the ADC_20_ provided the highest accuracy. No associations between the mean ADC_b_, ADC_total_, ADC_slow_, ADC_fast_ and f values of PDAC and the tumor grade were found. However, tumors with low values for all of the multiple-b-values DWI derived parameters had a tendency to be at advanced stage.

To the authors’ knowledge, three studies investigated the potential associations between ADC values of PDAC and tumor grade [12, 24, 25]. Similar b values (0, 500 or 800 s/mm^2^) and the same field strength of 1.5-T for DWI experiments to measure ADC values with a mono-exponential model. Wang et al. reported significantly lower ADC in cases of PDAC that are poorly differentiated in comparison with well/moderately differentiated lesions [24]. However, no associations between ADC values of PDAC and tumor grade in other two studies were observed [12, 25]. As in the present study, we failed to observe a statistically lower ADC in PDAC of poorly differentiated in comparison with those of moderately differentiated lesions, which is consistent with the results of Rosenkrantz A.B. et al [12] and Hayano K. et al [25].It is possible that histological differences between cases included in the studies account for the discrepant conclusions under the given single maximal b-value (500 s/mm^2^) [12].

Recently, IVIM DWI have been studied for pancreatic lesions [9, 18, 30]. Although IVIM parameters have been shown to aid distinguishing tumors from normal tissue, there is no work that compared IVIM parameters for the histological grade of tumors. The current results indicated that all of the mean monoexponential ADC (ADC_b_ and ADC_total_) and biexponential IVIM parameters (ADC_slow_, ADC_fast_ and f) values for PDAC did not exhibit significance dependence on tumor grade or tumor characteristics. Thus, based on the present data, it seems that the quantitative parameters are currently unlikely to be of clinical values for the non-invasive prediction of adverse pathological features of newly detected cases of PDAC.

Four research groups reported the IVIM-based parameters measurements in PDAC [9, 18, 20, 30–33]. Klauss M et al. reported the ADC_b_ of PDAC obtained from monoexponential model ranging from 4.04 to 1.18 × 10^−3^ mm^2^/s [30], which is in good agreement with our study. In addition, in the current study, the mean ADC_total_ values of PDAC is 1.37 × 10^−3^ mm^2^/s, which is also in good agreement with two previous studies (1.28 and 1.31 × 10^−3^ mm^2^/s, respectively) [13, 30]. Inconsistent with previous studies [11, 18], the perfusion fraction f was unable to distinguish PDAC from healthy pancreas. The main reason is that the IVIM DWI derived parameters are usually affected by the number and distribution of b values and post-processing methods used.

In addition to pathological factors that may impact ADC values, MRI technique itself including field strength, method for respiratory compensation, parameter variance and ADC measurement technique also influenced ADC measurements. In the present study, we found a significant decline of the mean ADC values of the monoexponential DWI from b_20_ to b_1000_ for the PDAC or the healthy pancreatic tissue. The mean ADC_b_ values were significantly lower for PDAC than for healthy pancreatic tissue except ADC_800_. Some previous studies showed significant difference in ADC_800_ values between PDAC and normal pancreatic tissue at 1.5-T [18, 25], the underlying reason for no significant difference in ADC_800_ at 3T as observed in this study maybe the variations in the data acquisition [33]. We also found that the ADC_20_ provided the highest accuracy to distinguish PDAC from healthy pancreas. It is necessary to optimize the low b values to differentiate pancreatic diseases in future studies, despite the perfusion effect on ADC values were obvious.

The present study had some limitations. Firstly, the number of subjects was limited, as many cases were PDAC with moderate differentiation or stage T3/T4. Further studies with larger samples size are needed to confirm our results. Secondly, our previous study had clarified that the effect of age and gender on ADCs in the normal adult pancreas can be excluded [34]. In the current study, we did not take into account the effect of age and gender on the ADC values of the control group, which may affect the results. Thirdly, in the current study, 58.8% of cancers were located in the pancreatic head. In these cases, it is difficult to find any normal tissue to compare with for there is obstruction of the pancreatic duct, which leads to significant atrophy of the rest of the pancreatic gland. So we did not analyze the DWI derived parameters of the PDAC tissue versus adjacent pancreatic parenchyma. Fourthly, for IVIM DWI, a navigator-triggered technique was employed to achieve higher SNR and decrease motion artifacts. Despite that the participants recruited were required to perform regular breathing training prior to scanning to decrease the misalignment between images, image registration at different b-values was not performed, which may affect the results. Finally, our findings were similar to the results of Rosenkrantz A.B. et al [12], but inconformity with the results of Wang et al[24]. It is possible that histological differences between cases included in the two studies account for the discrepant conclusions.

## Conclusions

Our results demonstrate that there were no associations between multiple-b-values DWI derived monoexponential and biexponential diffusion parameters of PDAC and tumor grade or tumor characteristics, and ADC_20_ provided the best accuracy for differentiating PDAC from healthy pancreas. This finding suggests that the clinical use of multiple-b-values DWI derived parameters to predict the prognosis of newly diagnosed PDAC is not advisable.

## Abbreviations

ADC: Apparent diffusion coefficient
AUC: Area under curve
CT: Computed tomography
DWI: Diffusion-weighted magnetic resonance imaging
ERCP: Endoscopic retrograde cholangiopancreatography
EUS: Endoscopic ultrasonography
IVIM: Intravoxel incoherent motion
LAVA: Liver acceleration volume acquisition
MRI: Magnetic resonance imaging
PDAC: Pancreatic ductal adenocarcinomas
ROC: Receiver operating characteristic
ROI: Region of interest
SPIR: Selective presaturation with inversion recovery
WHO: World Health Organization
